# The Influence of Transactive Memory System on Individual Career Resilience: The Role of Taking Charge and Self-Promotion

**DOI:** 10.3390/ijerph16183390

**Published:** 2019-09-12

**Authors:** Yuhao Liu, Xingchi Zhou, Shudi Liao, Jianqiao Liao, Zhiwen Guo

**Affiliations:** 1 School of Management, Huazhong University of Science and Technology, Wuhan 430074, China; Yuhao_liu@hust.edu.cn (Y.L.); Jimliao@hust.edu.cn (J.L.); 2 School of Management, Wuhan Textile University, Wuhan 430073, China; 3 Business School, Hubei University, Wuhan 430062, China; Guozhiwen@hubu.edu.cn

**Keywords:** transactive memory system, career resilience, taking charge, self-promotion, proactive behavior

## Abstract

The transactive memory system is known as an effective group cognitive system as well as a knowledge-sharing structure for organizations to keep competitive advantages in today’s dynamic and knowledge-based business environment. However, its influence at the individual level remains vague. The purpose of this study is to explore the influence of a transactive memory system (TMS) on individual career resilience through the theoretical perspective of conservation of resources theory (COR). This research proposes and examines a moderated mediation model that elaborates how a transactive memory system affects individual career resilience. A two-stage empirical study was conducted among 328 employees from companies in China. The findings suggest that a transactive memory system significantly influences individual career resilience positively, and employee taking-charge behavior plays a mediating role in that relationship. Furthermore, the results supported our moderated mediation model, which indicates that individuals with high self-promotion motives are more likely to engage in taking-charge behavior than those with low self-promotion motives, and the former reported higher career resilience than the latter eventually. Theoretical and practical implications are also provided in the discussion section.

## 1. Introduction

For the past decades, organizations have been getting increasingly dependent on teams or groups in order to be more productive, agile, and sustainable within the dynamic and challenging business world. Scholars and practitioners began to realize that successful utilization of the knowledge and expertise of each employee will promote performance improvement [1]. Recently, many researchers claimed that the transactive memory system (TMS) is an effective mechanism of incorporating employees with different expertise and coordinating their decentralized personal skills and knowledge. The transactive memory system (TMS) refers to a collective system that exists in teams or groups as a form of knowledge repository for encoding, storing, and retrieving information [2,3], and the system provides (1) information about each member’s specialized skills and expertise, and (2) a transactive processes that enable group members to link and cooperate with each other with their specialized expertise [4]. The concept of the TMS was initially developed in dyadic relationships, such as couples [5], and gradually extended to the organizational level and group level to explain team and organizational success. TMS as a social cognition system enables the organizations or groups to optimize the assignments of tasks to the appropriate group member as well as knowledge consultation and sharing. Some scholars found that the transactive memory system (TMS) offers efficiency and innovation [6] through which organizations can maintain a sustainable competitive advantage [7,8]. Thus, TMS has been more and more recognized and adopted in organizations [9], and has become an emerging area of academic research. Prior studies found that TMS contributes to team performance [10,11,12], team effectiveness [1], and team reflexivity [13]. The study of TMS has also been extended to the organizational level. Heavey and Simsek suggested that there is a positive relationship between a top management team’s TMS and firm performance [9]. Argote and Ren’s research indicated that TMS is positively related to organizations’ capabilities in dealing with the dynamic business environment [8]. Although most studies focused on the team level and the organizational level impact of TMS, some scholars also explored the influence of TMS at the individual level. For instance, Jarvenpaa and Majchrzak found that TMS would affect an individual’s ability to combine knowledge from others [14]. However, there is still relatively insufficient study focused on the influence of TMS on individual-level outcomes. Indeed, with a well-developed TMS, individuals are assigned tasks that best match their specific expertise and also aware of everyone else’s expertise; even if they had issues that are out of their specialties, they know whom they need to go for consultation [4]. Hence, TMS leads to great coordination, and job–person fit would eventually lead to positive outcomes of individuals. Thus, exploring the effect of TMS on an individual level would extend our understanding about the implications of TMS in organizations.

The current study is designed to fill the research gap on an individual level. We focused on the individual perception of TMS and examined how this perception might predict their behaviors and career-related capabilities in the longer term. The first goal of this study is to explore whether the TMS positively impacts individuals’ career resilience (CR). CR captures the ability to adapt to changing and even unfavorable environments in one’s career life [15,16]. As the work environment in today’s world becomes more dynamic and challenging, CR becomes an important factor for individuals to obtain a sustainable career [17] as well as occupational health, and CR as a type of career capability is developable [18]. Some studies suggested that CR increases with age and the accumulation of work experience [15,16], and career-management training programs may also enhance personal CR [19]. A study conducted by Abu-Tineh indicated that learning in organizations was positively associated with CR [20]. Drawing on the conservation of resource theory (COR) and in the view of existing literature on TMS and CR, we argue that by activating effective resource transition and investment, TMS will promote individual proactivity and self-confidence toward their job, and increase their CR eventually.

In addition, this study further explores the potential mechanism between TMS and CR by adopting COR as the theoretical foundation and focusing on the mediating role of an employee taking charge. Taking charge refers to one’s initiative and constructive efforts aiming at changing the status quo and facilitating organizational effectiveness [21]. As mentioned above, TMS is positively related to team effectiveness, team flexibility, and general performance, due to the effective task assignments and knowledge sharing within such a social system. Thus, we suggest that in an organization or group with well-developed TMS, it is most likely that members possess a bigger and clearer vision of what is going on in their work and have more confidence in making their personal work—even the whole work process of the group—more efficient and innovative. In other words, employees are more inclined to conduct taking-charge behavior in a more flexible, transparent team environment such as teams with great TMS. As a form of challenge-oriented proactive behavior [21], taking charge might lead to increased self-efficacy. At the same time, the change-oriented nature of taking charge makes it is always accompanied by risk while individuals conduct such behavior [21,22]. Scholars also suggest that voluntary engaging in taking charge indicates that individuals are less dependent on external instruction [21]. Taking together, taking-charge behavior is closely related to the three subdomains of CR: self-efficacy, risk taking, and dependency [15]. Thus, we presume that individuals with well-developed TMS are more inclined to perform taking charge-behavior, which would afterward contribute to increased CR.

The third purpose of this study is to examine the boundary conditions under which TMS results in elevated CR. In this study, we take self-promotion as an important moderator for the relationship between TMS and taking-charge behavior. Self-promotion behavior, as one strategy of image management, refers to individuals’ ability to anticipate being recognized as competent and valuable via showing off or talking about their experiences or accomplishments [23]. Individuals with high self-promotion are more likely to seek out any chance to demonstrate their competence [24]. Thus, we presume the individual differences of self-promotion would be a potential moderating factor. The high tendency of self-promotion would strengthen both the linkage between TMS and taking charge, as well as the indirect effect of TMS on CR.

To summarize, our research contributes to the transactive memory system (TMS) and career resilience (CR) literature in several ways. First, by applying the resource conservation perspective, our research sheds light on the effect of TMS on individual CR, which extends the outcome scope of TMS to include the individual level. Second, by testing the mediating role of taking charge, our research offers a novel mechanism by which TMS impacts CR. Finally, by examining the moderating role of self-promotion, our research identifies the boundary conditions for TMS to influence taking charge, which in turn influences CR. To test the hypotheses and research model, we conduct time-lagged two-wave survey research to better illustrate the relationships between the research constructs.

## 2. Theoretical Background

### 2.1. TMS and Career Resilience

To some extent, a transactive memory system (TMS) describes how individuals rely on each other to learn, communicate, and share knowledge in the group [3,25]. A well-developed TMS includes three characteristics: specialized knowledge and skills; an environment of mutual trust; and the transactive processes [26]. Specifically, team members have their various areas of specialized knowledge and skills, and they understand the knowledge map of the group and confidently rely on one another, which in turn facilitates them to work effectively and in a coordinated manner. By integrating members’ different knowledge structures, TMS enables teams and organizations to optimally allocate tasks and maximize the utilization of everyone’s expertise [27]. A number of researches have shown that TMS can lead to better team performance [28,29,30] as well as personal capabilities [14].

By conceptualizing TMS from the perspective of conservation of resources theory (COR), we aim to build a clear link between TMS and CR. COR indicates that all the valued entities can be viewed as resources, and individuals always strive to obtain, retain, protect, and foster different kinds of resources [31]. In this sense, TMS can be treated as a valued organizational resource for individuals, because it offers a trustworthy and knowledge-sharing environment, reduces the cognitive burden, and increases coordination for accomplishing tasks. According to COR theory, individuals in the workplace must put resources, such as the TMS, into a skill or competency development, and also need to protect against potential resource loss or gain more resources [32].

Career resilience (CR) was first introduced with career identity and career insight as components of career motivation by London [15]. CR is identified as the ability to resist career disruption and adapt to changing environments, even when the circumstance is less than optimal [15,33]. Due to the modern trend of many organizations downsizing and restructuring, practitioners have gradually noticed and accepted the importance of CR [34]. CR as an important personal resource [35] has been found to be related to many personal outcomes in the workplace in many studies. Hudgins’ survey study about nurses found that their career resilience is positively related to job satisfaction [36]. Another research conducted with the same sample of nurses suggested a positive relationship between resilience and career success [37]. Furthermore, scholars found that CR is not unchangeable, and organizations need to assist individuals to develop it [18,34]. Evidence showed that individuals who are willing to take risks, have less of a need for approval by others and hold high self-esteem or self-efficacy are more likely to have greater CR than others [15,38]. According to Hodges et al.’s research, personal fit with professional work and organization, as well as close relationships with others and optimized interaction with the environment can lead to a higher level of individual CR [39]. Also, CR is positively related to individuals’ relative job effectiveness and trust in other members [40].

As a kind of job resource, TMS is closely related to personal resources [41]. Specifically, TMS produces mutual trust and a high level of information sharing [42,43]. Thereby, whenever individuals need information, they can directly ask the right member for help [25], and access to relevant knowledge that will help them solve problems more quickly and easily. Such positive environments would largely reduce their hesitation in finishing tasks and in taking some risks to optimize their work, and thereafter enhance individuals’ CR level [44]. Core self-evaluation captures how individuals perceive themselves and their functioning in the world [45]. With a well-developed TMS, individuals can more easily improve their abilities, work in close coordination, and eventually complete tasks effectively, even if the work environment is dynamic and changing. Individuals with such a positive working environment are more likely to perceive valuable experiences work in collective situations and build high self-confidence and acknowledgment [46], which will eventually lead to a high level of CR perception [47]. As argued above, we posit that the individuals with access to the resources within a TMS are more career resilient.

**Hypothesis 1 (H1).**  *TMS is positively related to career resilience.*

### 2.2. The TMS and Taking Charge

As a motivational theory, conservation of resources theory (COR) suggest that individuals always strive to obtain, retain, foster, and protect resources, and they also get stressed or burned out when they lose resources or are threatened with loss or failure to get them. Within the COR theory framework, scholars suggest that people would always actively invest resources to gain more resources [32]. As mentioned above, taking charge can be considered as a method of personal resources investment, which involves voluntarily making constructive efforts to result in a functional change in the organization; this behavior is characterized as self-initiated, change-oriented and challenge-oriented [21]. Evidence shows that taking charge is positively related to performance [48,49]. However, scholars mentioned that taking-charge behavior also involves risks and costs [21], similar to all kinds of investment. The main reason is that change is not always welcomed in groups or organizations. Challenging the status quo may provoke skepticism and resistance among people; thus, taking charge may be viewed as inappropriate or even a threat, and the potential failure of change could lead to image loss or reputation damage. According to Parker et al.’s research, people are driven by “can-do” and “reason-to” motivations to perform proactively [50]. Specifically, “can-do” focuses on expectancy—that is, the perception of self-efficacy or self-evaluation would drive the employee to be more proactive, while “reason-to” underlines valence, such as self-determination or self-interest. Combing this perspective and COR, we expect that TMS is positively associated with taking charge via both “can-do” and “reason-to” motivations.

First, TMS is positively related to team effectiveness [51] and collective efficacy, which may have a salient influence on individuals’ cognitions such as self-efficacy and self-evaluation, and then impact individual “can-do” motivation. TMS offers the opportunity for individuals to access more useful information and utilize their special expertise and skills better in tasks, which would be helpful to increase individual self-efficacy and self-evaluation. According to the literature, individuals with high self-efficacy will be more likely to engage in behaviors related to taking charge [21]. In addition, TMS can reduce the risks of this behavior. With well-developed TMS, individuals can be aware of each other’s expertise and strength, and this would help them to trust each other more [52]. Therefore, when individuals attempt to challenge the status quo by taking charge, it is more likely to be regarded as discretionary and credible, and to be less resisted by others. Meanwhile, taking charge usually will lead to changes related to work methods, policies, and procedures in teams or organizations; thus, appropriate cooperation and coordination from other members are needed. On the other hand, there might be unintended consequences during the process of change [53]; individuals might need help from other members to face such circumstance. Thus, it is good to have TMS that helps individuals know how to coordinate their expertise with others, and easily get advice [52]. With the TMS, knowledge is shared via identifying the knowledge and expertise required for taking charge [54]; thereby, with the effective sharing of knowledge and skills, and good coordination in exercises, taking charge is guaranteed to be on the right trajectory and eventually lead to optimized results. In this sense, TMS enhances self-efficacy and reduces perceived risks, and then affects individuals to perform proactively.

Second, TMS can be considered as a valued job resource for individuals. Based on COR, the motivation for investing resources to gain more could provide individual “reason-to” perform proactively. According to the literature, TMS as a knowledge and human capital resource pool is associated with team performance [55] and team innovation [56]. In addition, TMS provides a trustworthy and knowledge-sharing environment, and such a trust relationship and effective information exchange quality would lead to high individual creativity [57]. According to COR, TMS helps individuals allocate and utilize their resources more effectively and efficiently through a supportive environment with mutual trust, as well as presents the opportunity to access more information provided by the TMS [9]. All these will reduce personal stresses and help employees to be more proactive in investing resources to gain preferable results. Previous research has also established that proactive behavior is associated with job performance [48] and job satisfaction [58]. For example, Cangiano et al.’s research found that individuals who perform proactively at work are more likely to perceive higher levels of competence and vitality [59]. Thus, individuals may engage in taking charge to get better performance assessments. Taken together, we hypothesize the following:

**Hypothesis 2 (H2).**  *TMS is positively related to taking charge.*

### 2.3. The Mediating Role of Taking Charge

TMS provides a supportive workplace for employees with increased CR, so it is important to identify the mechanism through which TMS affects CR [18]. In this study, we propose that taking charge serves as a channel via individuals’ cognition and attitude when the TMS translates into individuals’ CR. Taking charge is a spontaneous extra-role behavior, and it is not formally requested by organizations. It involves updating or modifying work methods, procedures, or policies in for individuals’ job, teams, or organizations. As taking charge provides opportunities to develop technical skills, individuals might gain perception of control and mastery via behaving proactively, and then develop self-efficacy and competence [60]. Scholars found that individuals who behave more proactively at work are more likely to perceive daily higher levels of competence and vitality [59], which we argue will eventually enhance CR. This is consistent with previous research indicating that self-efficacy and competence positively influence CR [19]. Meanwhile, Mishra and McDonald suggested that optimistic and self-directed attitudes may reduce the feeling of helplessness and hopelessness, and are positively associated with CR [18]. When people behave proactively, they perceive increased job satisfaction [58] and obtain higher performance appraisal [48]. Such good experiences can lead to positive and optimistic attitudes toward work and help individuals to be less risks-averse and more likely to attempt new things. Thereby, we argue that taking charge could promote CR via a positive attitude.

Furthermore, as a form of work exploration and learning behavior, taking charge exerts challenges into routine jobs, such as using new methods or correcting faulty procedures. These challenges provide individuals with the opportunity to be creative and take risks, and then they are more likely to gain high levels of CR [16,33]. Given that taking charge is change-oriented and aiming at challenging the status quo, and is usually accompanied by unpredictable risks, individuals who conduct such behavior need to communicate with others to get support and coordinate every factor to reduce risks and achieve goals [61]. Such contingencies and opportunities for updating professional and managerial skill will increase the individual level of CR [62]. To sum up, the preceding discussion indicates that TMS provokes taking-charge behavior, which in turn reinforces CR. Thus, we expect that taking charge mediates the impact of TMS on CR. That is, the TMS will elevate individuals’ CR at least partly because individuals perform taking-charge behavior when motivated by TMS. Thus, we hypothesize the following:

**Hypothesis 3 (H3).**  *Taking charge mediates the relationship between TMS and career resilience.*

### 2.4. The Moderating Role of Self-Promotion

In the past decades, sociologists and social psychologists have found that individuals usually attempt to influence the image that others have of them by using different strategies [23,63]. The concept of impression management captures the differential processes through which individuals enhance their self-image [64]. Scholars found that impression management is prevalent in organizational settings [65], and the impression management tactics can be categorized into five categories: ingratiation, exemplification, intimidation, self-promotion, and supplication [66]. Some scholars suggested that impression management can motivate individuals to perform proactively [23]. Moreover, individuals are significantly different in choosing their impression-management strategies [67]. In the current study, we focus on self-promotion strategy, which is more about individuals wanting to be considered as competent. Self-promotion refers to an impression-management strategy whereby individuals demonstrate their abilities or achievements in order to be seen as competent by others [24].

Taking charge offers a good opportunity for individuals to demonstrate their competence and value to others, for it entails voluntary and constructive efforts by individuals to update their work method, improve their work procedures, and create policies for the jobs, teams, or organizations [21]. With all these characteristics, individuals who successfully conduct taking-charge behavior are viewed as valuable and competent by others. For this reason, engaging in taking-charge behavior meets the goal of impression management, especially for those who are engaging in a high level of self-promotion. Thus, individuals know that they can directly and effectively impress and demonstrate their competence and value to others deeply by taking charge. Furthermore, scholars have suggested that individuals usually will take advantage of outside opportunities to meet their inner needs [68]. As discussed above, TMS is a valuable resource. Self-promoted individuals who possess resources such as a TMS are more likely to engage in self-initiated activities, which will enable them to be recognized by others. TMS provides incentives and opportunities for individuals to engage in taking-charge behavior and make them feel confident that they can bring about change successfully. With the support of a TMS, individuals with a high level of self-promotion are more prone to conduct taking-charge behavior. By contrast, individuals with low self-promotion are less motivated to show off experience or competence, and they are less likely to be motived by TMS to engage in extra-role behavior. Thereby, compared with individuals with low self-promotion, the positive effect of TMS on taking charge is more significant for the ones who have high self-promotion motives. Drawing from the above discussion, we argue that the influence of TMS on taking charge is moderated by self-promotion. Compared with individuals who have low self-promotion, individuals with high self-promotion motives are more inclined to invest the resources provided by TMS into extra-role behavior, and are more likely to take charge. Thus, we predict that:

**Hypothesis 4a (H4a).**  *Self-promotion moderates the relationship between TMS and taking charge such that the relationship is stronger for higher self-promoting individuals than it is for lower self-promoting individuals.*

According to the argument of Hypothesis 3 and Hypothesis 4a, we further propose that self-promotion may moderate the relationship of TMS on individual CR via taking charge, thereby leading to a moderated mediation. Taking charge influenced by TMS has a positive relation with CR, and self-promotion positively moderates the process through which TMS impacts CR. Specifically, for high self-promoting individuals, the relation of TMS to CR is stronger, and accordingly, the indirect effect of taking charge on the relationship between TMS and CR is increased. By contrast, for low self-promoting individuals, the effect of TMS on CR will be weaker, and accordingly, the mediating effect that taking charge had on the relationship between TMS and CR will be reduced. Thus, we propose that:

**Hypothesis 4b (H4b).**  *Self-promotion moderates the mediating effect of taking charge on the relationship between TMS and CR such that this effect is stronger for higher self-promoting individuals than it is for lower self-promoting individuals.*

**Figure 1:**
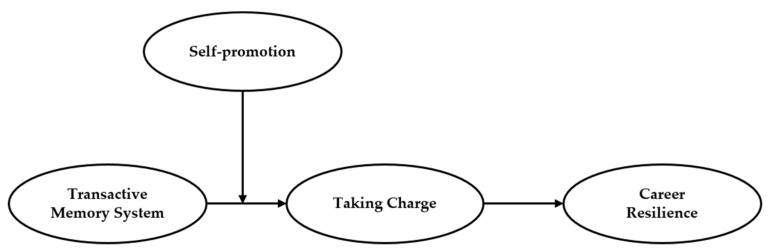
Hypothesized model of the processes linking a transactive memory system (TMS) to career resilience (CR) through taking charge and self-promotion.

Our hypothesized model is shown in Figure 1 as follows.

## 3. Method

### 3.1. Sample and Procedures

The data of this survey was collected from 13 different types of companies located in mainland China cities (including Wuhan, Yichang, Shenzhen, Guangzhou, Dongguan, Shanghai, Nanjin, and Hangzhou), which include state-owned companies and private companies. The industries include manufacturing, information technology (IT), biotechnology, and the healthcare industry (see Table 1). We chose Chinese firms as our ideal research sample for two reasons. Firstly, the knowledge management of Chinese firms have been noticed by both scholars and practitioners more and more as their influences increases in the world knowledge economy. Second, the above-mentioned industries that we studied in China are heavily reliant on knowledge management and innovation as well as on the cooperation of groups and teams to gain competition advantages. We conducted two waves of data collection with a six-week time lag between the first and second data collection in 2018. With the help of the human resources (HR) department head of each company, we were able to assign a numerical code to each of the participants, so that we could match the data from the two time stages. In the first wave of data collection, the participants were asked to report their demographic information as control variables such as gender, age, education, the length of tenure in the organization, as well as their evaluation of TMS, taking charge, and self-promotion (time 1). Six weeks later, participants were asked to complete a survey measuring CR (time 2).

We distributed 500 questionnaires, and 435 questionnaires were returned, yielding an 87% response rate. In the second wave, 355 out of 435 questionnaires were returned, yielding a response rate of 81%. After taking out inappropriate (e.g., all items scored the same) and incomplete questionnaires, a total of 328 valid cases were used for further analysis. We looked into the demographic statistics of the missing 80 participants in the second round of the survey; they had no particular features than other being participants, which indicates that this is just a normal loss of sample (see Table 1 and Table 2). Within the final samples we adopted, 60% were male, and 40% were female. Their average age was 37 years. More than half (55.8%) of the participants had a bachelor’s degree, 10.4% had a master’s degree or higher, 29.9% had a junior college education, 4.0% had high school education or below, and their average length of organizational tenure was 7.85 years.

We also would like to note that this research did not involve any human clinical trials or animal experiments as well as any unethical behaviors during the whole data collection process. The ethics board of Huazhong University of Science and Technology approved this study. The participants in this study signed written consent before they completed the survey.

### 3.2. Measures

All the scales used in these studies were originally written in English. Thus, we conducted translation and back-translation procedures [69] to create Chinese versions of all the scales. All the variables were measured on five-point Linkert scales, and individuals were asked the extent to which they agreed with the statements (1 = strongly disagree, 5 = strongly agree).

**Transactive memory system (TMS).** A 10-item scale developed by Akgun et al. was used in this study [70]. Participants were asked to assess the impact of the TMS in their group interaction by three dimensions. Sample items are: “Each team member has specialized knowledge of some aspect of our project” for the specialization dimension, “I was comfortable accepting procedural suggestions from other team members” for the credibility dimension, and “We accomplished the task smoothly and efficiently” for the coordination dimension (Cronbach’s α = 0.89).

**Taking charge.** Following previous research [71], the four highest factor-loading items were adopted from Morrison and Phelps’ original scale to assess the participants’ taking charge in this study [21]. Sample items are: “This person often tries to institute new work methods that are more effective for the company”, and “This person often tries to bring about improved procedures for the work unit or department” (Cronbach’s α = 0.80).

**Career resilience.** The seven-item measure of CR presented by Day and Allen was adopted to measure the participants’ feeling of their career-resilient ability in the second time stage [33]. Sample items are “I am willing to take risks” and “I am able to adapt to changing circumstances” (Cronbach’s α = 0.84).

**Self-promotion.** Self-promotion motives were measured by Bolino and Turnley’s five-item scale [24]. Example items are “Talk proudly about experience or education” and “Make people aware of accomplishments” (Cronbach’s α = 0.88).

**Control variables.** We controlled the demographic variables such as gender, age, and education level. We also controlled the length of tenure in the current company. These control variables were found to have a significant relationship with career motivations such as career resilience [33,72].

## 4. Results

### 4.1. Confirmatory Factor Analyses, Descriptive Statistics, and Correlations

In the current study, we conducted confirmatory factor analysis (CFA) with Mplus 7.4 to examine the discriminant validity of the main variables. We first assessed the six-factor model that included TMS (three dimensions), taking charge, career resilience, and self-promotion. This model had quite an acceptable fit with indexes (χ^2^ = 534.62, df = 260; CFI = 0.92; TLI = 0.91; RMSEA= 0.057; CFI: Comparative Fit Index; TLI: Tucker-Lewis Index; RMSEA: Root Mean Square Error of Approximation.).

Then, we examined the model fit of four alternative models to compare with the hypothesized six-factor model. In model 1, three dimensions of TMS make up the same factor (χ^2^ = 903.94, df = 269; CFI = 0.82; TLI = 0.80; RMSEA = 0.085).

In model 2, TMS and taking charge make up the same factor (χ^2^ = 1252.64, df = 272; CFI = 0.72; TLI = 0.69; RMSEA = 0.105). Model 3 combined the three variables collected in the first wave, including TMS, taking charge, and self-promotion (χ^2^ = 1860.18, df = 274; CFI = 0.55; TLI = 0.51; RMSEA = 0.133). In model 4, all the items of four variables were combined into one (χ^2^ = 2469.68, df = 275; CFI = 0.38; TLI = 0.32; RMSEA = 0.156). The results indicate that our hypothesized six-factor model had better model fit indexes than the other alternative models (*p* for Δχ^2^ < 0.01); thus, the discriminant validity of the main constructs used in the current study was supported.

Table 3 presents the descriptive statistics, intercorrelations, and reliability for the study variables.

### 4.2. Measurement Model

We first adopted hierarchical multiple regression to test Hypothesis 1. We controlled for demographic characteristics, and the results revealed that TMS has a positive relationship with individual CR (b = 0.22, *p* < 0.01), supporting Hypothesis 1.

Then, we adopted the PROCESS macro for SPSS developed by Hayes [73] to assess the hypotheses involving mediating, moderating, and moderated mediation effects (Hypotheses 2–4). During the regression analysis, a bootstrapping method was implemented to enlarge the sample to 5000, and all the statistical significance of the effects we tested was computed based on bias-corrected confidence intervals. Table 4 shows the results of regressions testing Hypothesis 2 and Hypothesis 3 using bootstrapping in SPSS PROCESS macro.

Hypothesis 2 proposed that TMS would be significantly related to taking charge. The result of Model 1 shows that TMS is positively and significantly related to taking charge (b = 0.51, *p* < 0.01, Model 1). Thus, Hypothesis 2 is supported. After controlling for TMS and the control variables, taking charge was positively and significantly related to career resilience (b = 0.12, *p* < 0.01, Model 2). Moreover, the indirect effect of TMS on career resilience via taking charge was significant (b = 0.06, boot 95% CI [0.03,0.12]), supporting Hypothesis 3.

To examine the moderating role of self-promotion between TMS and taking charge, we centralized the data and conducted hierarchical regression. As indicated in Model 4 and shown in Table 5, self-promotion positively moderated the linkage of TMS and taking charge (b = 0.34, *p* < 0.01).

As showed in Table 6, conditional indirect effects of TMS on career resilience via taking charge were stronger when self-promotion was high (b = 0.09, boot 95% CI [.03, 0.16]), but when self-promotion was low, the conditional indirect effect was insignificant (b = 0.03, ns). These results supported Hypothesis 4b and identified that self-promotion strengthens the mediating effect of taking charge of the relationship between TMS and career resilience. These results revealed that self-promotion strengthens the mediating effect of taking charge of the relationship between TMS and CR. Thus, Hypothesis 4b was supported.

**Figure 2:**
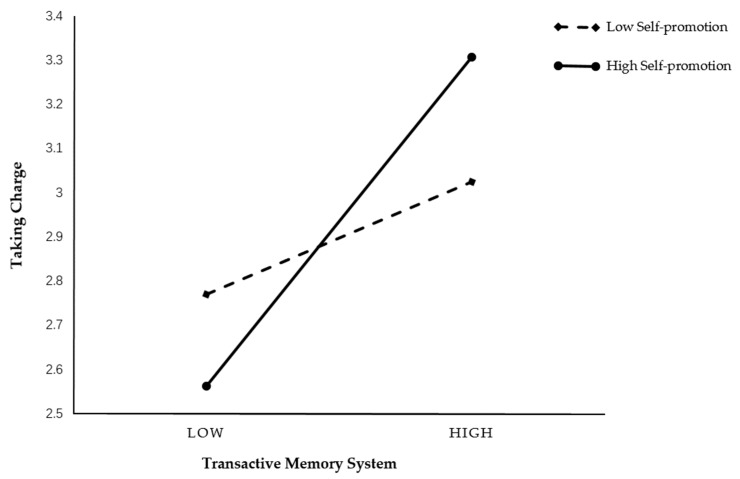
Interaction effect of TMS and self-promotion on taking charge.

To better illustrate the interaction patterns, the simple slopes were plotted (see Figure 2). The figure showed that the forms of interaction consisted of our prediction that the relationship between TMS and taking charge was stronger when individuals had a high level of self-promotion than when they had a low level of self-promotion. Taken together, Hypothesis 4a was supported.

## 5. Discussion and Conclusions

The present research investigated the effect of TMS on an individual level through the resource-based perspective. We theoretically proposed and empirically examined whether TMS motivates individuals to perform proactively (i.e., exhibiting taking charge), and eventually translates into personal CR. Moreover, the relationship between TMS and taking charge and the mediating effect of taking charge for this linkage were moderated by self-promotion. That is to say, for individuals with high self-promotion motives, TMS will more effectively prompt behaviors related to taking charge; also, the mediating effect on TMS and CR relationship is strengthened.

In sum, TMS as a social cognitive system can be utilized as an effective resource and competitive advantage for teams and organizations. Therefore, it is important for both scholars and practitioners to get deeper insights on how and when TMS will positively affect individuals. Our theoretical model and empirical results extend th literature of TMS by exploring the mechanism of how TMS encourages individuals to perform proactively and whether this change is correlated with their CR. We hypothesized that TMS positively influences CR, and taking charge mediates this influence. Moreover, we identify self-promotion as an important boundary condition factor for the above relationships. We hope our work improves the current understanding of TMS and inspires future studies.

### 5.1. Theoretical Contributions

Our research contributes to the transactive memory system (TMS), taking charge, and career resilience (CR) literature. For the first and biggest contribution, by adopting conservation of resources theory (COR) as the theoretical foundation, we have proposed and examined a research model that focuses on the potential effect of the TMS on individual CR. Although previous research has found that TMS can benefit teams and organizations [8,10], the relationship between TMS and individual-level outcomes has not been examined. However, the TMS as a social cognitive system that depends on individual knowledge structures as well as the coordination of individual expertise [4] can also be considered as a resource within the group or organization. In this case, the development of the TMS determines how much one can utilize it as a resource to invest in career advance according to the conservation of resource perspective, by which we can claim that TMS will influence individual-level outcomes. Thus, we theoretically and empirically identified the influence of TMS at the individual level. By introducing the resource-based perspective, our research offers a fundamental theoretical justification for the function of TMS. The result supports our anticipation that TMS can contribute positively to individual-level constructs of CR. Such a finding substantially extends the scope of the studies on TMS’s outcomes by including an important form of personal ability. This finding also demonstrates that as a critical contextual resource, the value of TMS is not limited to the team and organizational level, but it is also functional at an individual level.

Meanwhile, our research has enriched the literature on CR as well. Past research primarily focused on the effect of workplace support on CR [74,75]. However, the effect of a supportive environment provided by the knowledge system on CR has seldom been discussed and examined. CR is an important ability for employees to adapt to today’s changing and challenging career environment [76], and this ability is finite and developable [18]. Our founding indicates TMS as a unique and effective form of resource that can be translated into individual career resilient capacity.

In addition, our findings regarding the mediating role of taking charge also offer deeper insights into the research on the linkage between TMS and CR in general and with TMS in particular. First, by introducing the resource-based perspective, our research explicitly explains the transmitting processes from TMS to CR via individuals performing proactively, which complements the scarcity of research on the underlying mechanism by which the environment is associated with individual CR [18]. This finding suggests that individuals with a resource provided by TMS are more inclined to make voluntary and constructive efforts in the workplace, and such work experience consequently translates into CR. Second, as noted earlier, although many studies have claimed the critical impact of TMS on teams and organizations, an insufficient amount of research has focused on the effect that TMS has had on the individual level. By applying the COR perspective, this study focused on individual perceptions of TMS, and our findings highlighted that TMS motivates individuals to engage in taking charge, and then, as a form of proximal behavioral outcome of TMS, taking charge can help to transmit the influence of TMS on CR.

Furthermore, this study extends the current literature by testing the moderation effect of self-promotion on the relationship between TMS and CR via taking charge. Specifically, the indirect effect of TMS on CR via taking charge was found to become stronger when self-promotion was higher compared with the lower end. These findings suggest that the processes involved in transmitting a well-developed TMS to CR through taking charge seem to be more effective and beneficial for individuals with high self-promotion motives. At the same time, by integrating TMS and the image management perspective, this study deepens our understanding of how collective cognition systems, individual proactive extra-role behaviors, and CR relate to each other.

### 5.2. Practical Implications

For practitioners, our study offers a comprehensive understanding of how TMS can be translated into individuals’ CR partially through engaging in taking charge, which offers guidance to both practitioners and organizations. This is important because CR has been positively associated with organizational commitment as well as job satisfaction [77], which then lead to less intention to quit [78]. Moreover, organizations are also facing a more competitive and challenging environment nowadays, and increasing the proactivity of their employees can help organizations gain developmental and competitive advantages [79]. Thus, as organizations are expecting employees to take charge and be more career resilient, building TMS might be an important strategy. Organizations and group leaders need to develop a well-functioning TMS, which may encourage employees to be more proactive (e.g., conduct taking-charge behavior) and result in enhanced CR. On the other hand, the contemporary career environment is dynamic and stressful for individuals, so there is a need for them to develop career resilience to cope with increasing career uncertainty [35]. For employees who want to be more competent and resilient in an uncertain and even disruptive environment, a TMS may provide opportunity and help to them. Employees who experienced well-developed TMS could expect to be more capable of dealing with different situations in work, and eventually benefit a certain group or organization.

In addition, the current research demonstrates that individuals with relatively high-level self-promotion motives are likely to translate the benefits of TMS into taking charge, which in turn enhances CR. Although image management motives have been seen as inappropriate, individuals who want to be viewed as valuable may be truly dedicated to their organizations [23]. Therefore, this study suggests that managers need to be moderately tolerant of employees’ self-promotion behaviors, which would promote individuals to behave proactively and obtain increased CR.

### 5.3. Limitation and Future Directions

This study still suffers several research limitations, which would be considered in the future. Firstly, we adopted a longitudinal design to collect the data at two time stages, which may address some the causal logic concerns about our hypothesized model. However, we measured all the variables using the employee’s self-rating source. We need to consider multiple sources for the data in a future study, especially considering a non-self-rating source for the behavioral outcomes of the employee. To take out the worries about the reliability and validity of our results in this study, we chose our survey design for the following reasons: Previous research pointed out that taking charge can be better measured when self-reported [71], and we controlled for tenure and demographic variables. Besides, the results of Harman’s one-factor analysis showed that six extraction components had eigenvalues over 1, and the sum of squared loading of the first factor is only 23.53%, indicating that the fits of Harman’s one-factor analysis were unacceptable [80]. Taken together, we believe that our research is not seriously threatened by common method variance. Another concern is that there was only a six-week span between our two-stage survey, which might be too short to capture the change of an individual’s career attitudes and capabilities. Even though this study showed significant associations between our proposed variables, studies with longer time spans are still needed to construct a model with more reliability. Also, our study suffers from a limited sample size, which is the traditional barrier of many clinical experiments and research studies. Acquiring large samples in future studies will help to strengthen the generalizability of the results.

Second, as a form of resource, TMS can elicit motivation for taking charge, and then be translated into an individual resource as CR. However, it is worthwhile and needed for further studies to explore the effect of TMS on the individual level. For instance, TMS provides an environment of mutual trust and knowledge-sharing, based on social exchange theory, such an environment could be helpful to enhance individual affective commitment to the organization and reduce quit intentions.

In addition, in the current study, we focused on the moderation role of self-promotion. Comparing with other forms of impression-management motivations, individuals who have high self-promotion motives focus more on showing off qualifications and competence. However, it could still be interesting for future studies to examine the moderation role of different image management strategies.

Last but not least, even though many studies adopted Chinese firms as their research settings and samples [81,82], we still need to pay attention to the cultural effects as we try to generalize our finds. In the most recent study of Bachrach et al. [56], they explored cultural effect using meta-analysis and found that some cultural contexts such as power distance and in-group collectivism can influence the TMS and performance relationships. In the future study, adopting multi-cultural samples may be helpful to generalize our finds. Moreover, it would be interesting to look into the cultural unique constructs such as ‘Guanxi’, which may have a great impact on TMS and the TMS–performance relationship. Guanxi refers to personalized social ties and networks that not only involve work and exchange-oriented relationship (e.g., leader–member exchange relationship) but also involve non-work related connections (e.g., mutual interest, caring for personal life) [83]. Guanxi can be established either between leaders and members or between group members, and there can be many dyadic relationships as well. The quality of Guanxi within a group may have a profound influence on the dynamics and interactions with TMS.

## Figures and Tables

**Table 1:** Description of investigated and industries and firms; valid cases in each time stage.

Industry	Firms Included	By Percentage	Valid Cases in Time 1	Valid Cases in Time 2	Loss of Cases in Percentage
1. Manufacturing	2	15%	110	84	24%
2. IT	5	39%	146	102	30%
3. Biotechnology	3	23%	91	72	23%
4. Healthcare	3	23%	88	70	21%

**Table 2:** Description of dropping out participants.

	Mean	S. D.	Details
1. Gender	1.53	0.46	57% males, 43% females
2. Age	36.58	5.31	
3. Education	2.78	0.85	11% with master’s degree or higher; 53% with bachelor’s degree, 31% with junior college education; 5% with high school education or below
4. Tenure	7.36	7.22	

**Table 3:** Means, standard deviations, and correlations among variables.

	Mean	S. D.	1	2	3	4	5	6	7	8
1. Gender	1.40	0.49	1.00							
2. Age	37.02	5.69	0.15 **	1.00						
3. Education	2.73	0.71	−0.01	−0.02	1.00					
4. Tenure	7.85	6.52	0.02	0.26 **	−0.22 **	1.00				
5. TMS	4.09	0.48	−0.11 *	0.02	−0.04	0.00	1.00			
6. Taking Charge	3.46	0.76	−0.06	0.11	0.00	0.12 *	0.33 **	1.00		
7. Career Resilience	3.81	0.51	−0.19 **	−0.08	−0.01	−0.07	0.22 **	0.22 **	1.00	
8. Self-Promotion	3.02	0.79	−0.17 **	−0.02	0.06	0.04	0.09	0.07	0.30 **	1.00

Note: N = 328, * *p* < 0.05, ** *p* < 0.01, Gender was dummy-coded as 1 (= male) and 2 (= female). TMS: transactive memory system.

**Table 4:** Regression results for testing the mediation effect.

	Dependent Variable			
	Taking Charge		Career Resilience	
**Predictor Variable**	**Model 1**		**Model 2**	
	**B**	**S.E.**	**B**	**S.E.**
**Controls**				
Gender	−0.06	0.08	−0.16 **	0.06
Age	0.01	0.01	−0.01	0.01
Education	0.04	0.06	−0.02	0.04
Tenure	0.01	0.01	−0.01	0.00
**Independent Variable**				
TMS	0.51 **	0.08	0.16 **	0.06
**Mediator**				
Taking Charge			0.12 **	0.04
**R^2^**	0.13		0.11	
**ΔR^2^**	0.10 **		0.03 **	
**F**	9.52 **		6.75 **	

Note: * *p* < 0.05, ** *p* < 0.01. The b values are unstandardized regression coefficients. TMS: transactive memory system.

**Table 5:** Regression results for testing moderation effect.

	Dependent Variable			
	Taking Charge			
**Predictor Variable**	**Model 3**		**Model 4**	
	**B**	**S.E.**	**B**	**S.E.**
**Controls**				
Gender	−0.05	0.08	−0.03	0.08
Age	0.01	0.01	0.01	0.01
Education	0.04	0.06	0.06	0.06
Tenure	0.01	0.01	0.01 **	0.01
**Independent Variable**				
TMS	0.50 **	0.08	0.52**	0.08
**Moderator**				
Self-Promotion	0.03	0.05	0.04	0.05
**Interaction**				
TMS×Self-Promotion			0.34 **	0.10
**R^2^**	0.13		0.16	
**ΔR^2^**	0.10 **		0.03 **	
**F**	7.99 **		8.71 **	

Note: * *p* < 0.05, ** *p* < 0.01. The b values are unstandardized regression coefficients. TMS: transactive memory system.

**Table 6:** Conditional indirect effects of TMS on CR via taking charge.

Moderator	Level	Effect	Boot S. E.	Boot 95% C.I.	
				LL	UL
**Self-Promotion**	High	0.03	0.02	0.01	0.08
	Low	0.09	0.03	0.03	0.16

Note: Bootstrap 5000.
